# Infinite mixture-of-experts model for sparse survival regression with application to breast cancer

**DOI:** 10.1186/1471-2105-11-S8-S8

**Published:** 2010-10-26

**Authors:** Sudhir Raman, Thomas J Fuchs, Peter J Wild, Edgar Dahl, Joachim M Buhmann, Volker Roth

**Affiliations:** 1 Department of Computer Science, University of Basel, Bernoullistr. 16, CH-4056 Basel, Switzerland; 2 Department of Computer Science, ETH Zurich, Universitaetstrasse 6, CH-8092 Zurich, Switzerland; 3 Competence Center for Systems Physiology and Metabolic Diseases, Schafmattstr. 18, CH-8093 Zurich, Switzerland; 4 Institute of Pathology, University Hospital Zurich, Schmelzbergstrasse 12, CH-8091 Zurich, Switzerland; 5 Institute of Pathology, University Hospital Aachen, Pauwelsstrasse 30, 52074 Aachen, Germany

## Abstract

**Background:**

We present an infinite mixture-of-experts model to find an unknown number of sub-groups within a given patient cohort based on survival analysis. The effect of patient features on survival is modeled using the Cox’s proportionality hazards model which yields a non-standard regression component. The model is able to find key explanatory factors (chosen from main effects and higher-order interactions) for each sub-group by enforcing sparsity on the regression coefficients via the Bayesian Group-Lasso.

**Results:**

Simulated examples justify the need of such an elaborate framework for identifying sub-groups along with their key characteristics versus other simpler models. When applied to a breast-cancer dataset consisting of survival times and protein expression levels of patients, it results in identifying two distinct sub-groups with different survival patterns (low-risk and high-risk) along with the respective sets of compound markers.

**Conclusions:**

The unified framework presented here, combining elements of cluster and feature detection for survival analysis, is clearly a powerful tool for analyzing survival patterns within a patient group. The model also demonstrates the feasibility of analyzing complex interactions which can contribute to definition of novel prognostic compound markers.

## Background

Survival Analysis is a branch of statistics dealing with the analysis of time-to-failure data and is applicable to a variety of domains like biology, engineering, economics etc. More generally, it is the analysis of time-to-event data where an event could signify death, failure etc. Particularly in the context of disease studies, it is a powerful tool for understanding the effect of patient features on survival patterns within a group. A parametric approach to such an analysis involves the estimation of parameters of a probability density function which models time. The model is further extended by considering the effect of covariates (X) on time via a regression component. Cox’s proportionality hazards model, as explained in [1], is a popular model for modeling such an effect:

 (1)

where* h*_0_(*t*) is the baseline hazard function (chance of instant death given survival till time *t*), *x* is the vector of covariates and *β* is a vector of regression coefficients. In this paper, we focus on covariates which are categorical in nature, since it is a frequently encountered case in biological applications.

In the past, such models have been extended to a mixture model (mixture of survival experts) in order to find sub-groups in data with respect to survival time along with measuring the effect of covariates within each sub-group. In this context, (Rosen and Tanner) [2] define a* finite* mixture-of-experts (MOE) model by maximizing the partial likelihood for the regression coefficients and by using some heuristics to resolve the number of experts in the model. A more recent attempt at this analysis, which was carried out by [3], uses a maximum likelihood approach to infer the parameters of the model and the Akaike information criterion (AIC) to determine the number of mixture components. A Bayesian version of the mixture model has been investigated by [4], which analyzes the model with respect to time but does not capture the effect of covariates. On the other hand, the work by [5] performs variable selection based on the covariates but ignores the clustering aspect of the modeling. Similarly, [6] defines an infinite mixture model but does not include a mixture of experts, hence assuming all the covariates to be generated from the same distribution and also assumes a common shape parameter for the Weibull distribution.

In this paper, we unify the various important elements of this analysis into a Bayesian infinite mixture-of-experts (MOE) framework to model survival time, while capturing the effect of covariates and also dealing with an* unknown* number of mixing components. The number of experts are inferred using a Dirichlet process prior on the mixing proportions, which overcomes the issue of deciding the number of mixture components beforehand [7]. The regression component, introduced via the proportionality hazards model, is non-standard since the Weibull distribution is not part of the exponential family of distributions due to the lack of fixed-length sufficient statistics. Another novel feature of this framework is the addition of sparsity constraints to the regression coefficients *β* in order to determine the key explanatory factors (covariates) for each mixture component. Since the covariates are discrete in nature, each variable is transformed to a group of dummy variables and sparsity is achieved by applying a Bayesian version of the Group-Lasso (as described in [8] and [9]) which is based on a sparse constraint for grouped coefficients [10]. We demonstrate the ability of the model to recover the right sparsity pattern with simulated examples. In a related work, [11] show sparsistency (sparse pattern consistency) of the lasso in the limit of large observations. The following sections describe all the components of this unified framework with some results on a breast-cancer dataset.

## Methods

In this section, we explain the overall model in an incremental way starting first with a regression model for survival analysis and then attaching a clustering model to it. This also highlights the incremental nature of the algorithm presented for inference.

### Bayesian survival regression

To begin with, we focus on defining a single cluster model. For survival analysis, we model the distribution of a random variable *T* (representing time) over the interval [0, ∞). Further, a standard survival function is defined based on the cumulative distribution over *T* as follows:

 (2)

which models the probability of an individual surviving up to time* t*_0_. The hazard function *h*(*t*), the instantaneous rate of failure at time *t*, is defined as follows:

 (3)

For modeling purposes, our choice of distribution for modeling time is the Weibull distribution which is flexible in terms of being able to model a variety of survival functions and hazard rates. Apart from flexibility, it is also the only distribution which captures both the accelerated time model and the proportionality hazards model, see [12] for details. The Weibull distribution is defined as follows:

 (4)

where* α_w_* and λ*_w_* are the shape and scale parameters, respectively. Based on the above definition and assuming right-censored data (see [1] for details), the likelihood can be written as:

 (5)

where *N* is the number of observations, *δ_i_* = 0 when the *i^th^* observation is censored and 1 otherwise. Further, to model the effect of covariates *x* on the distribution over time, we apply Cox’s proportional hazards model. Under this model, the covariates are assumed to have a multiplicative effect on the hazard function:

*h*(*t*|*x*) = *h*_0_(*t*) exp(*f*(*x*, *β*)),

(6)

where* h*_0_(*t*) is the baseline hazard function, *x* is the vector of covariates and *β* is a vector of regression coefficients. In our model, we assume the function *f* to be a linear predictor i.e. *f*(*x*, *β*) = *η* = *x^T^β*. We also consider higher-order interactions (first-order - pairs of features, and second-order - triplets of features etc.) instead of modeling just the main effects (individual features). Further flexibility is added to the linear predictor by adding a random effect in the following manner:

*η* = *x^t^β* + ∈, where ∈ ~ *N*(0,*σ*^2^).

(7)

The likelihood is modified as follows to include the covariate effect:

 (8)

We note that although most parts of the model described so far resemble an enhancement of a generalized linear model (GLM) (see [13]) called a random-intercept model, it is not strictly a GLM since the Weibull distribution lacks fixed-length sufficient statistics and is not considered, in a strict sense, to be part of the exponential family of distributions unless the shape parameter is known. Although the Weibull distribution lacks fixed-length sufficient statistics, for the two parameters (*α_w_*, *λ_w_*), it is still possible to define a joint conjugate prior ([14]), as is explained in the subsection on priors eq. (10). In order to provide a full Bayesian treatment of the model, we define suitable conjugate priors for the other parameters of the model, namely* σ* and *β*.

#### *Contrast coding*

**Figure 1:**
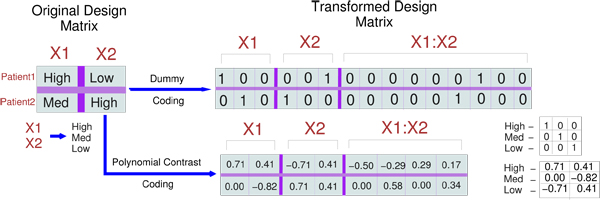
**Dummy coding illustration**. On the top-left, categorical observations for 2 patients are shown for whom 2 biomarkers (*X*1 and *X*2) are measured for expression values. Each biomarker (categorical variable) can have three possible values (high, med and low). The top-right side shows the transformed covariate matrix after the dummy coding procedure has been applied. The resulting design matrix represents each variable as a group of dummy-variables. Hence identifying key features from the original matrix is translated to the problem of identifying key* groups* of dummy variables. The bottom-right shows the transformed matrix after using a polynomial contrast coding procedure. The resulting contrast-coded matrix uses (*K* − 1)*^order^*^+1^ columns for an interaction as opposed to (*K*)*^order^*^+1^ columns in a a dummy-coded matrix where *K* is the number of categories for a variable and *order* denotes the order number of the interaction (zeroth, first, second etc).

In biological applications, it is very common to encounter categorical data. When the *x_i_*’s are categorical variables, a suitable coding procedure is applied to the variables (see standard textbooks like [15]) in order to obtain the design matrix for inference. Apart from single variables (interactions of order zero), the design matrix also consists of higher-order 1st order (pairwise interactions) and 2nd order (triplet interactions). An example of a two variable (with three categories) observation matrix with a first-order interaction transformed using dummy coding is shown in Fig. 1 (top). A default dummy coding procedure leads to over-parametrization (redundancy in the number of columns) and this effect becomes profound with greater number of levels and higher-order interactions. Also in many biological applications, the categorical variables have a natural ordering in the values that they take, for example - intensity values. Based on these requirements, we use polynomial contrast codes since they are suited for ordered categorical variables and avoid over-parametrization by representing a *K*-level variable with* K*−1 columns (see Fig. 1 (bottom)). This results in representing each categorical variable as a group of contrast-coded variables. Hence, to create the full design matrix, first the levels are contrast-coded (using a standard R function) which gives us the codes for respective levels (see Fig. 1 (bottom-right)) and then each observation is recoded (for main effects and higher-order interactions) using these codes as reference.

#### *Priors*

One of the major requirements of the model is to find the key explanatory factors from data. To achieve this goal, we need to apply sparsity constraints on the regression coefficients *β* to identify the key interactions. As described, the coding procedure gives rise to groups of contrast-coded variables. This transformation of data leads to the task of inferring sparsity on a group level, i.e. on* grouped* dummy variables, where each group represents a single variable in the original formulation.

Hence, for parameter *β*, we apply the general prior defined in [9] to a special case for Bayesian Group-Lasso (as defined in [8] for a Poisson model), which is suitable for sparse inference in grouped variables for the model that we have defined. The sparse prior is motivated by the classical Group-lasso which can be recovered in the log-space based on defining the prior as a product of Multivariate Laplacians. Although a direct representation of the prior exists, in order to make the posterior analysis feasible (to obtain standard conditional posteriors), we redefine the prior as a two-level hierarchical model, by introducing latent variables λ*_g_*. For the Bayesian Group Lasso, the hierarchical prior over the regression coefficients is defined as follows:

 (9)

where *G* is the number of groups,* p_g_* is the size of group *g*, *ρ* and *σ*^2^ play the role of the Lagrange parameter in classical Group-Lasso and each* β_g_* is a scaled mixture of Multivariate-Gaussians. Based on (9), we can derive the marginal pdf of* β_g_* analytically as a product of Multivariate Laplacians (for details, see [8]).

A full Bayesian treatment of the model is achieved by introducing a prior on *σ*^2^, based on a standard conjugate joint prior (see [16]), described as a product of a Normal distribution of *β* given* σ* and an inverse-chi square distribution of , and a conjugate Gamma prior on *ρ*. Although the Weibull distribution lacks fixed-length sufficient statistics, for the remaining two parameters (*α_w_*, λ*_w_*), it is possible to define a joint conjugate prior, as explained in [14]:

 (10)

where *a*,*b*,*c* > 0 and* d* allows us to deal with the lack of fixed-length sufficient statistics.

**Figure 2:**
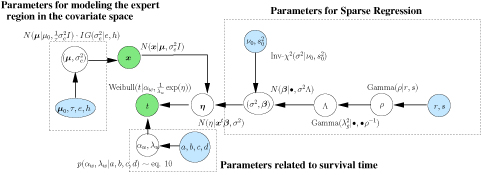
**Model description with all the parameters involved for a single cluster**. The complete hierarchical model with the parametrization for a single cluster model. Depicted in blue are the hyperparameters for the respective distributions, like (*r*, *s*) for the Gamma prior on *ρ*. The observed variables *x* denoting the covariates and *t* denoting time are shown in green. The part of the figure centered around *t* forms the core which defines the generalized linear model with a Normal random link between *η* and the covariates and coefficients and priors for the Weibull distribution. The block on the right defines the hierarchy related to the sparse regression on the covariates via the hierarchical representation of the Normal-Gamma prior on the regression coefficients *β*. Furthermore, the left block defines the variables for describing the distribution of the covariate space.

The full model with all the variables is described in Figure 2.

#### *Posteriors*

In practice, sampling from the posterior distribution will not be possible directly, hence we propose to use a Gibbs sampling strategy for stochastic integration. The sampling process further enables this procedure to be incorporated very naturally as another step in the clustering algorithm discussed in the next section. Additionally, for the lasso model, the Blocked-Gibbs sampler has been shown to be geometrically ergodic in [17]. Hence the convergence of the Gibbs sampler is expected to be very rapid. Multiplying the priors with the likelihood and rearranging the relevant terms yields the full conditional posteriors, which are needed in the Gibbs sampler for carrying out the stochastic integrations. The posterior for *σ*, *β*, *ρ* and  are exactly as defined in [8]. The conditional posterior of *η_i_* is difficult to sample from since it is not of standard form. However, since the conditional posterior is log-concave, we propose the use of Laplace approximation, similar to that in [18], which approximates the conditional posterior to a Normal distribution and simplifies sampling considerably. Although alternatives exist in the form of adaptive-rejection sampling, the Laplace approximation gives results that are indistinguishable while speeding up computations considerably.

For the Weibull parameters *α_w_* and λ*_w_*, sampling based on their individual posteriors conditioned on each other is avoided, since this results in a slow mixing of the Markov chain due to a high correlation between samples from the two conditionals. To overcome this issue, the conditional posterior of (α*_w_*, λ*_w_*) is split up into the conditional of λ*_w_* given* α_w_* which results in an Inverse-Gamma distribution,

 (11)

where *y* is the number of deaths (number of data points for which *δ_i_* = 1) and the marginal of* α_w_* which is derived based on the work in [14]:

 (12)

where *P_y_* is the product of *t_i_*’s for which *δ_i_* = 1 and (●) represents all the unknown parameters. This marginal results in a non-standard distribution, and sampling is done via a discretized version of the same.

### Infinite mixture of survival experts

**Figure 3:**
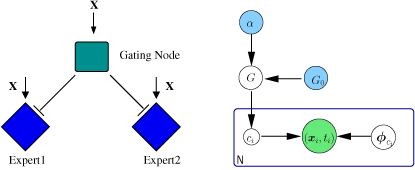
**Infinite Mixture-of-experts model. Left panel:** Mixture-of-experts model for two experts with a gating node representing the function that decides which of the two experts is chosen to make a prediction for *x* which is represented by *p*(*c_j_*|*x*, ●) in eq. (13). **Right panel:** Infinite mixture of experts using a Dirichlet process prior *G* with parameters (*α*, *G*_0_). *N* denotes the number of observations and *c_i_* the respective assignment variables. The observed variables *x* and *t* are represented in green with the priors collapsed to . In the full model, the  part will be replaced by Figure 1.

**Finite mixture of experts.** The previous section described the inference procedure when the data is assumed to be generated from one global group. We further enhance this idea by removing this assumption and model data which is potentially generated from multiple (and known number of) sub-groups/clusters in data. In order to model the clustering in terms of the combined effects of features *x* and survival time *t*, we use an MOE model as defined in [19] (see Figure 3: Left panel). It consists of a fixed number of experts, each expert explaining the distribution of time for a particular region in the covariate space. Hence the *t* based clusters or mixing components, represented by experts, are probability distributions conditioned on the covariates *x*. The distribution of *t* can be written based on a standard mixture model conditioned on *x* as:

 (13)

where (●) represents all the unknown parameters and* c_j_*’s are the mixture components. The first term in eq. (13) is the gate function which decides which *j^th^* expert is best suited for making a prediction for feature vector *x*. Using Bayes’ rule, we can rewrite the model in the following way in order to resemble a standard mixture model, as shown in [20]):

 (14)

This representation allows us to visualize each mixture component as a joint distribution over (*x*, *t*). The distribution over *x* is modeled as a Normal distribution  as show in Figure 2. The standard joint conjugate prior of Normal-Inv-*χ*^2^ is applied to the parameters . The posterior conditionals are also of standard form and hence can be easily incorporated into the Gibbs sampling scheme introduced in the previous section. To complete the Bayesian picture, we need to apply a suitable prior to the mixing proportions *c*. In a finite MOE model, a Dirichlet distribution is a standard conjugate prior to the mixing proportions. All other parameters and priors, based on the modeling of (*x*, *t*), follow from the previous section.

**Infinite mixture of experts.** The above model was described for the case when the underlying number of clusters is fixed/known. We now add the final enhancement to our model by removing this limiting assumption as well. The model is extended to an infinite mixture-of-experts by replacing finite clusters by infinite clusters and hence replacing the Dirichlet distribution by a Dirichlet process (DP) as prior for the mixing proportions, similar to [20]. The Dirichlet process is a distribution on distributions i.e. a particular sample from a DP is also a probability distribution from which samples can be drawn. The draws from a DP are discrete hence making it a useful prior for clustering purposes. In this manner, the effective number of clusters can be inferred from data by carrying out MCMC sampling from the posterior distribution. This model extension is described in a hierarchical manner as follows (see Figure 3):

 (15)

where DP denotes a dirichlet process prior with base distribution *G*_0_ and a concentration parameter *α*, *c_i_* is the latent class to which an observation (*x_i_*, *t_i_*) belongs and *ϕ_c_* denotes the parameters which determine the distribution of class *c*. Further hierarchy is added to *ϕ_c_* (parameters) by adding suitable priors as defined in Section 2.

**Algorithm 1:** **Algorithm 1** Blocked Gibbs Sampling for a Truncated Dirichlet process

1:	**Input**: N observations *D* = (*x_i_*, *t_i_*).
2:	**Initialize**: *c_i_* = random cluster assignments and parameters .
3:	Draw from the posterior of the joint distribution *p*(π, Φ*, *c*) by drawing from the conditionals.
4:	**while** NotCoverged **do**
5:	Sample Φ* | π, *c*, *D* - This is carried out individually for each parameter in the model conditioned on the rest.
6:	Sample *c* | Φ*, π, *D* - For *i* = 1,…, *N*, draw values , *c_i_* = 1,…, *M*.
7:	Sample π | Φ*, *c*, *D* - The mixture proportions are drawn based on the posterior *P*(π|*α*)*P*(*c*|π).
8:	**end while**

**Markov Chain Monte Carlo (MCMC) sampling for Inference and Parameter Estimation.** The inference of the infinite-mixture-of-experts model is carried out by MCMC sampling of the posterior distribution. Although there exist non-conjugate versions of the Dirichlet process algorithms (as given in [21]) which can be applied for inference, for practical reasons, we use a truncated version of the Dirichlet process called the Dirichlet-Multinomial allocation model [22], by specifying an upper bound on maximum number of clusters based on the prior knowledge of the particular application. It serves as a good approximation to the DP measure and results in a finite-sum random probability measure which is computationally easy to deal with and easy to implement. More specifically, we carry out a Blocked-Gibbs sampling on a truncated Dirichlet process (see Algorithm 1 for details). After initializing all the parameters, the sampling algorithm is executed till the point of convergence. The point of convergence can be determined based on the length-control diagnosis explained in [23] or fixed to a maximum number of iterations based on studying the traceplots of the sampling process in simulations.

## Results and discussion

**Simulations**. In order to demonstrate the effectiveness of the model, experiments were carried out on simulated data. The first experiment shows the capability of the model to correctly identify two sub-groups in data along with identifying the key explanatory factors in both groups. The dataset of size 150 was generated from two equally proportioned clusters with (5, 5) and (1,1) being the shape and scale parameters for the Weibull distribution for each cluster. The features consisted of 7 variables with expansion up to 2nd order interactions (63 terms). For the first cluster, the significant factors included main effects *X*1, *X*3 and *X*4, all first order interactions with these three variables i.e. (*X*1 : *X*3), (*X*1 : *X*4), (*X*3 : *X*4) and a second order interaction (*X*1 : *X*3 : *X*4). Similarly, for the second cluster, the significant factors included main effects *X*2, *X*6 and *X*7, all first order interactions with these three variables (i.e. (*X*2 : *X*6), (*X*2 : *X*7), (*X*6 : *X*7)) and a second order interaction (*X*2 : *X*6 : *X*7).

**Figure 4:**
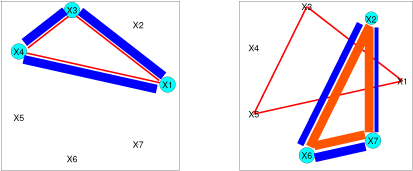
**Results for simulated data**: 2 clusters with 7 categorical variables having interaction terms up to second order. In all interaction graphs, the light-blue circles represent the main effects, the blue lines represent 1st-order pairs and the reddish triangular lines indicate 2nd-order triplet interactions. In each case, the size of the circle or the width of the lines indicates the estimated significance of the main effect or the higher-order interaction: i.e. For example on the right cluster, more than 90% of the posterior samples for variable 2 have a positive sign. Based on the results of the inference process, we observe that all the key features have been correctly identified.

Significance was achieved by assigning *β* values of (3, 3, 3, 3, 3, 3, 3) and (3, 3, 3, 3, 3, 3, 3) to the specific factors in the respective clusters and the rest of the *β* coefficients to zero. The covariates themselves were sampled from a Normal distribution with means (0.3, 0.3, 0.3, 0.3, 0.3, 0.3, 0.3) and (0.7, 0.7, 0.7, 0.7, 0.7, 0.7, 0.7) for each cluster respectively. The Gibbs sampling process was executed for 50,000 iterations and the burn-in was observed to be very early (in the first ≈ 100 iterations). Both the clusters were detected and all the true significant factors for both clusters were identified successfully. See Figure 4 for details.

**Figure 5:**
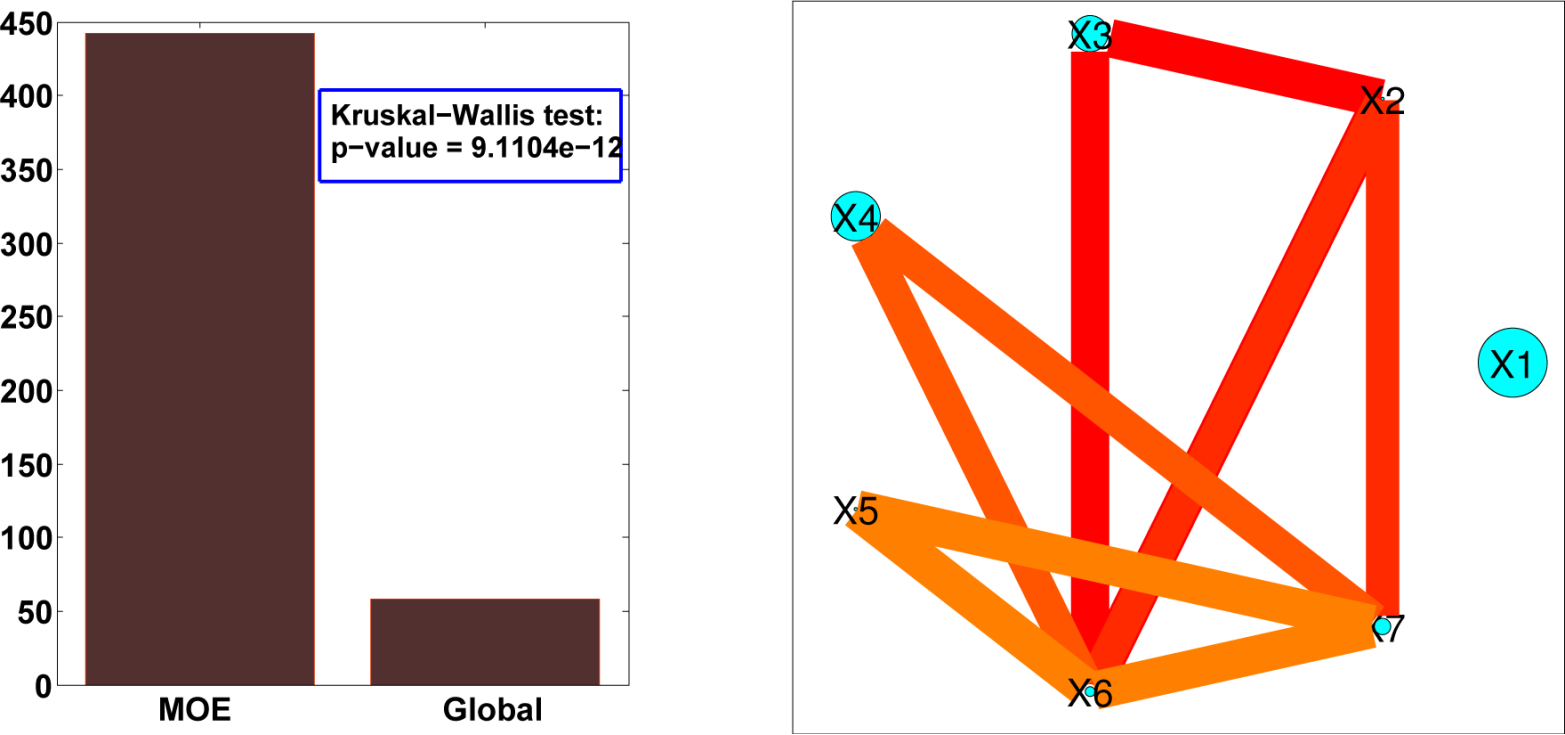
**Comparison to a global model. Left:** The actual number of points in the test set which scored better in a particular model (442 for MOE Vs 58 for Single Cluster) based on the likelihood scores. Results of the Kruskal-Wallis rank test also validated this observation with a p-value ≪ 0.001. **Right:** Results of the key interactions found for a single cluster model. Some of the key factors are not identified along with existence of many false-positives.

In the second experiment, we compare our mixture-of-experts model to a global single cluster model in order to justify the need for a mixture model. The training data generated in the first experiment was used again for learning the parameters of a single-cluster model. In order to compare the two models, a separate test set (of size 500) was generated additionally to evaluate the performance of both models by comparing the log-likelihood of all the test points based on the parameters learned by both models. The per-point comparison is shown in Figure 5 which indicates the improvement achieved by using a MOE model. We also performed a standard Kruskal-Wallis rank test which also ranks the MOE model higher than the single cluster model (see Figure 5 left panel). Apart from the quantitative evaluation, we also see in terms of identifying the significant factors (see Figure 5 right panel), that the single cluster model does poorly, both in recognizing the true factors and in terms of false positives. This can be explained based on the fact that in a single cluster model, the model has to assume a common baseline model (for both clusters). Then, in order to adjust for the real survival patterns, it can only achieve the same effect by making suitable adjustments to the regression component. In doing so, the model compromises in terms of the identification of significant factors from data. As a result, we see that the MOE model performs much better than a one-cluster model, hence justifying the need for a cluster-based model.

**Application to Breast-Cancer dataset.** The dataset consists of measured intensity levels obtained from tissue microarrays of the following markers: karyopherin-alpha-2 (KPNA2), nuclear staining for p53, the anti-cytokeratin CK5/6, the fibrous structural protein Collagen-VI, the inter-*α*-trypsin inhibitor ITIH5, the estrogen receptor (ER) and the human epidermal growth factor receptor HER2. From these categorical variables we constructed covariates arranged in a design matrix which includes all dummy-coded interactions up to the second order.

Cross-validation experiments were conducted for both the MOE and single cluster model which gave rise to similar trends but with unclear significance. Despite of the fact that this dataset is one of the biggest of its kind, the rather low number of samples (270 patients) remains the main challenge in these scenarios. A further difficulty is the large number of censored patients (60%), which is a common problem in long term retrospective studies.

**Figure 6:**
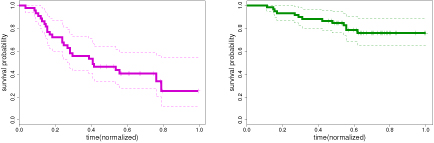
**Kaplan Meier plots for the identified sub-groups**. Kaplan-Meier plots for the high-risk group (left) and the low-risk group (right). The high-risk group contains a large number of patients, who die early.

**Figure 7:**
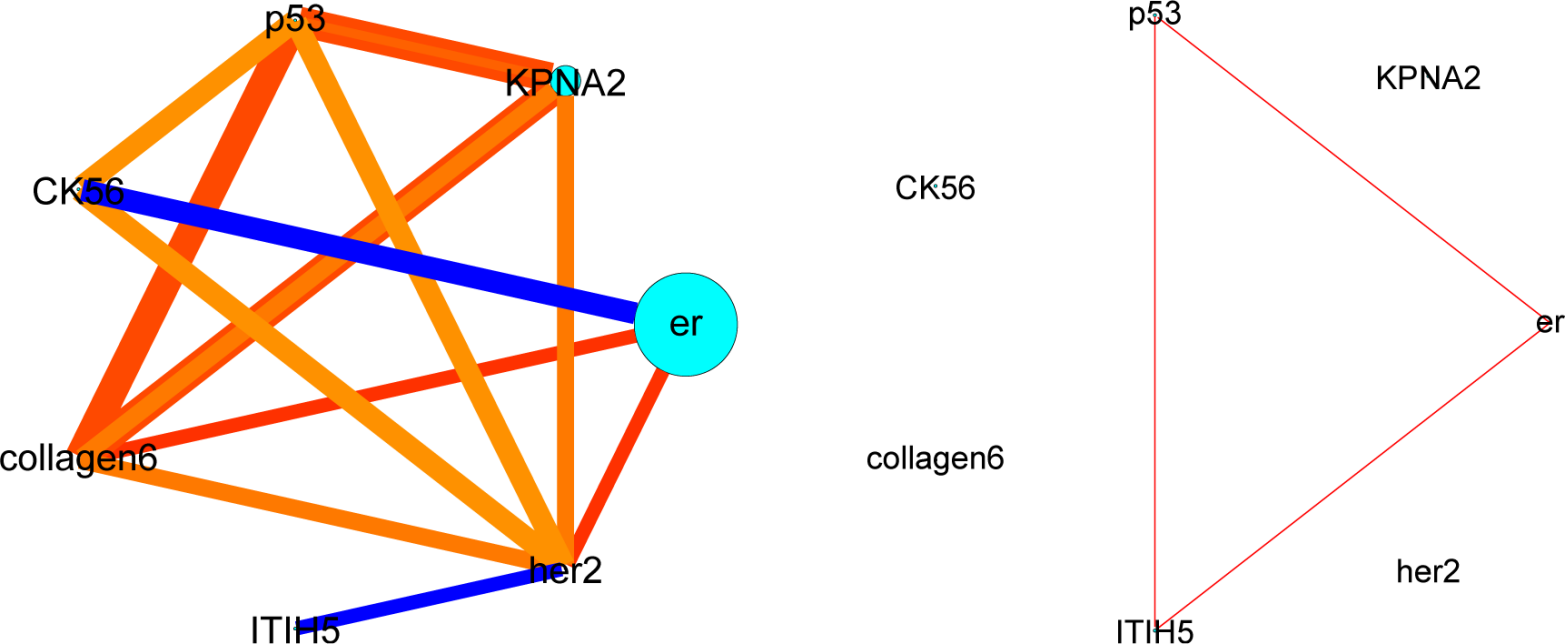
**Breast Cancer results - key interaction patterns for the identified sub-groups**. Identified interaction patterns for the high-risk group (left) and the low-risk group (right). The size of the circles indicates the estimated significance of the main effects. For instance, the largest circle for ER means that the 0.9 posterior quantile does not contain zero. Correspondingly, the line-width of the interactions (blue lines: 1st-order, reddish triangles: 2nd-order) indicates their significance.

Over a wide range of prior-values, the Dirichlet process mixture model for selecting “survival experts” finds two large and highly stable clusters. In order to externally validate these clusters, we analyze the survival of the underlying patient populations by way of classical Kaplan-Meier plots, see Figure 6. It is obvious that the survival experiences of patients belonging to the two clusters differ significantly, with cluster 1 basically containing all patients who die early. In Figure 7, the interaction patterns within the two clusters are shown as lines connecting pairs or triplets of markers, where the line-width encodes the significance in terms of posterior quantiles which do not contain zero.

The high-risk patient cluster is characterized by a global underexpression of ER and overexpression of basically all other markers, in particular KPNA2, CK5/6 and HER2. Overexpression of the latter two markers clearly identifies this cluster as a collection of* basal*- and HER2-type breast-cancer patients. The occurrence of KPNA2 in the high-risk group is also in accordance with previous studies: KPNA2 is a member of the karyopherin (importin) family, which is part of the nuclear transport protein complex. KPNA2 overexpression has been shown in several gene expression signatures in breast cancer and other cancer types. KPNA2 overexpression has been previously identified as a possible prognostic marker in breast cancer [24].

The group-Lasso detects several strong higher-order interactions. Interpreting these interaction terms can be a complex problem, but a close analysis of the contrast codes and the sign of the regression coefficients shows that the weak prognosis of members in this cluster is dominated by some of the combinations, details in Table 1 where ↘ means underexpression and ↗ overexpression.

**Table 1:** Interpretation of interaction terms

ER	↘				
ER	↘	CK5/6	↘		

KPNA2	↘	p53	↘	Collagen-VI	↘

ITIH5	↗	HER2	↗		

ER	↘	Collagen-VI	↘	HER2	↗

ER	↘	KPNA2	↘	ITIH5	↗

ER	↘	p53	↗	CK5/6	↘

ER	↘	KPNA2	↘	Collagen-VI	↘

The observation that high-order interaction terms seem to be even more indicative than the individual main effects is a highly interesting result of this study which may lead to the definition of novel prognostic markers for better differentiation between high-risk patients. Together with our medical partners we are currently testing these new hypothetical compound-markers.

The low-risk cluster has a clear* luminal*-type signature (strong ER response). Hardly any significant patterns can be identified which, however, is quite understandable by noticing that the survival curve is almost flat for these patients: in the proportional hazards model the individual covariates influence the “passage of time”, and a flat curve basically means that there is almost no intra-class variation that could be explained by individual covariate effects.

## Conclusions

We have introduced a fully Bayesian survival infinite mixture-of-experts model which extends classical approaches by including feature selection for contrast-coded categorical variables. Random links and a mixture-of-experts architecture allow for both stochastic and model-driven deviations from the underlying parametric survival model. The inherent clustering property of the final model makes it possible to identify patient groups which are homogeneous with respect to the predictive power of their covariates for the observed survival times. The built-in Bayesian feature selection mechanism reveals cluster-specific explanatory factors and interactions. Due to the Bayesian treatment within a suitably expanded model, posterior samples can be generated efficiently which makes it possible to assess the statistical significance based on a very large number of draws.

Applied to survival data from a breast cancer study, the model identified two stable patient clusters that show a clear distinction in terms of survival probability. Several strong high-order interactions between marker proteins were detected which carry more information about the survival targets as the markers themselves. Not only does this result confirm earlier studies, it also shows that the analysis of complex interactions is feasible and may lead to the definition of novel prognostic markers. We are currently conducting new experiments to test these new hypothetical compound-markers.

## Authors contributions

SR, TJF, JMB and VR have contributed toward designing the model and drafting the manuscript. PJW and ED are domain experts in pathology and molecular biology and have contributed with respect to conducting biological experiments, generating the required samples and in analyzing the results, i.e. estimating the protein expression on the immunohistochemical stained slides. All authors read and approved the final manuscript.

## List of abbreviations

AIC: Akaike information criterion; MOE: Mixture of experts; GLM: Generalized linear model; MCMC: Markov chain Monte Carlo; DP: Dirichlet Process

## Competing Interests

The authors declare that they have no competing interests.
